# Dissecting the Genetic Correlations and Causal Effects of Gut Microbiota on Pan‐Cancer Phenotype: Driven by Common Dietary Preferences

**DOI:** 10.1002/fsn3.70708

**Published:** 2025-07-28

**Authors:** Yuhang Zhou, Yun Feng, Yue Wang, Yiwen Wang, Xiaolin Liu, Tao Sun, Junnan Xu

**Affiliations:** ^1^ Department of Breast Medicine 1 Cancer Hospital of China Medical University, Liaoning Cancer Hospital Shenyang China; ^2^ Department of Pharmacology Cancer Hospital of China Medical University, Liaoning Cancer Hospital Shenyang China; ^3^ Department of Bioinformatics Kanghui Biotechnology Co., Ltd. Shenyang China; ^4^ Department of Breast Medicine Cancer Hospital of Dalian University of Technology, Liaoning Cancer Hospital Shenyang China

**Keywords:** causal effect, dietary preference, genetic correlation, gut microbiota, pan‐cancer

## Abstract

Previous studies have found that the gut microbiota are associated with various types of cancer. However, the effects under the pan‐cancer phenotype and whether they are driven by dietary preferences remain unclear. Here, we aim to comprehensively examine the genetic correlations and causal effects between gut microbiota and pan‐cancer using linkage disequilibrium score (LDSC) regression and Mendelian randomization (MR). In addition, MR was also used to estimate the causal effects of dietary preferences on pan‐cancer and gut microbiota. The causal steps approach and two‐step MR will be used to construct the mediation network. After Bonferroni correction, we identified three gut microbiota that showed suggestive genetic correlations with pan‐cancer. In addition, our evidence suggests that six gut microbiota provide suggestive causal effects for pan‐cancer. However, in the evidence for dietary preferences on pan‐cancer, we identified four significant effects and 20 suggestive effects. Seven pairs of causal effects were identified in the MR analysis of dietary preferences on gut microbiota. After excluding unrobust effects in the sensitivity analysis, we identified four mediation pathways through the causal steps approach. These findings will help us further understand the role of gut microbiota and dietary preferences in cancer and provide valuable insights into dietary patterns for populations at risk of cancer.

## Graphical abstract

Cancer is one of the leading causes of death worldwide. Our study indicates that common dietary preferences can influence pan‐cancer risk by driving changes in gut microbiota composition. We recommend that populations at risk of cancer consider appropriate probiotic or dietary interventions. valuable insights into dietary patterns for populations at risk of cancer.

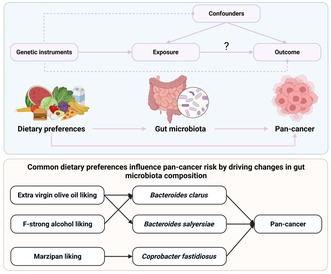

## 1 Introduction

Cancer is one of the leading causes of death worldwide (Bray et al. 2024). The complex interactions between gut‐resident microbiota and the host have introduced a new realm of knowledge in tumorigenesis, cancer progression, and treatment (Su et al. 2024; Teng et al. 2023; Yu et al. 2024). Although early researchers have investigated the diversity and composition of the gut microbiota in individual tumors as much as possible, it remains largely underexplored under the pan‐cancer phenotype. This may be influenced by the limitations of the data and the interference of confounders.

In the context of cancer biology, the effects of the gut microbiota and its metabolites on the host are both pro‐cancerous and anti‐cancerous (Zhou et al. 2024). In recent years, several bacterial species have been associated with tumorigenesis and cancer progression. *Helicobacter pylori* ‐induced gastritis is considered the strongest driving factor for gastric cancer (Polk and Peek 2010). Furthermore, the cooperation between *Lactobacillus johnsonii* and *Clostridium sporogenes* offers a new adjunctive approach to improve the tumor microenvironment (Jia et al. 2024). Given the potential of the enormous gut microbiota in cancer, researchers have proposed probiotic supplementation and fecal microbiota transplantation to restore the diversity of the gut microbiome. However, emerging evidence suggests that dietary modifications are more effective than probiotic supplementation alone in regulating the gut environment (Yin et al. 2025). Therefore, understanding diet‐driven changes in the gut microbiota may lead to dietary recommendations and interventions aimed at reducing cancer risk.

However, the long duration and high costs of observational studies limit our hope of quickly acquiring new knowledge in this field. Therefore, a non‐traditional method was hoped to quickly reveal new findings to drive further research. Recently, previously published genome‐wide association study (GWAS) summary statistics have been used to evaluate the genetic correlation and causal effect between traits. Linkage Disequilibrium Score (LDSC) regression can assess genetic correlation from GWAS data. Mendelian Randomization (MR) can infer causal effect using genetic variants as instrumental variables (Weng et al. 2023; L. Chen et al. 2025; He et al. 2025). Since genotype precedes phenotype and alleles are randomly allocated at conception, this allows genetic variants to serve as instrumental variables to effectively avoid bias, confounders, and reverse causality interference. Then, we assessed the genetic correlation and causal effect of the gut microbiota on the pan‐cancer phenotype in this study. Meanwhile, two‐step MR estimated the dietary preferences driving gut microbiota and the pathway effect.

## 2 Method

### 2.1 Study Design

**FIGURE 1:**
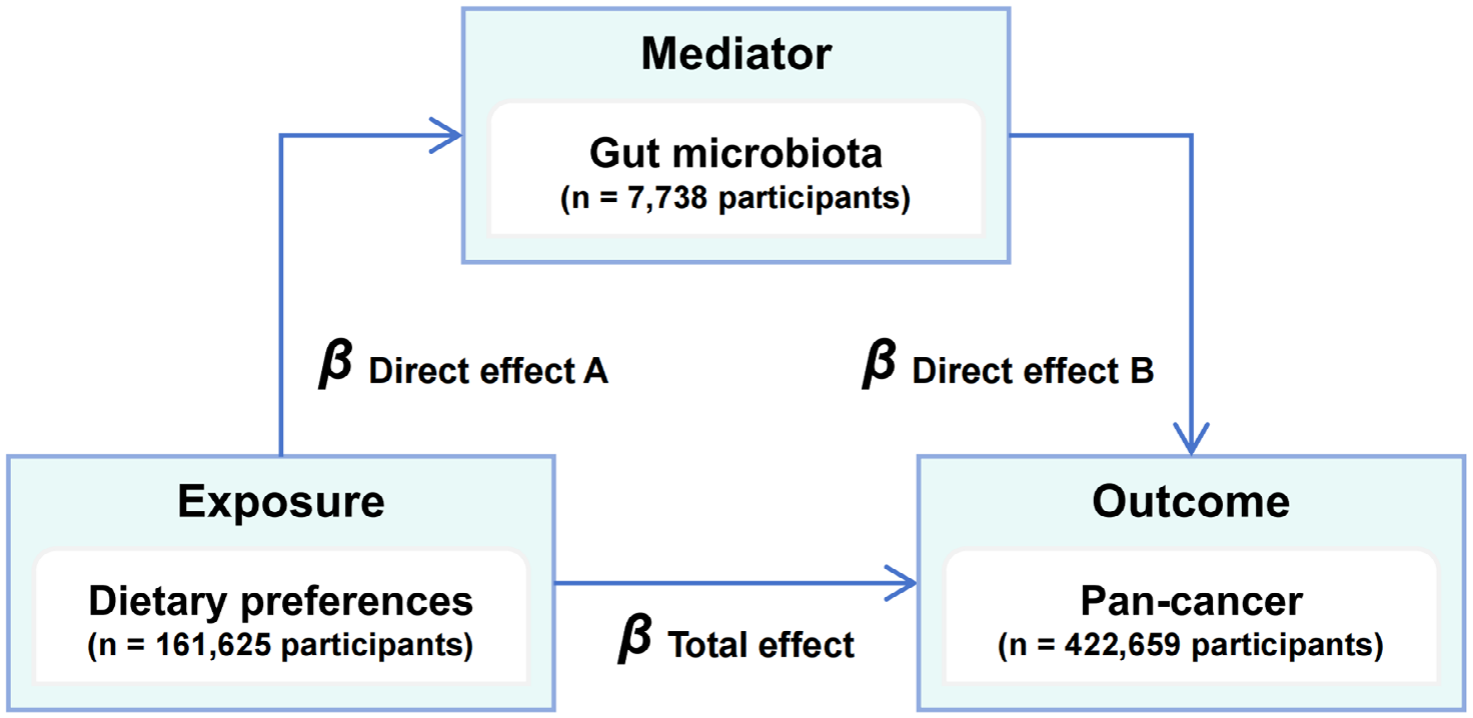
Overview of study design.

We performed LDSC regression and MR analysis to estimate the genetic correlation and causal effect of the gut microbiota on pan‐cancer phenotype. The further two‐step MR analysis estimated the dietary preferences driving the gut microbiota from a genetic perspective. The detailed workflow of this study is shown in Figure 1. This study follows the Strengthening the Reporting of Observational Studies in Epidemiology‐MR (STROBE‐MR) guidelines (Table S1) (Skrivankova et al. 2021).

### 2.2 Data Source

The GWAS summary statistics of the species‐level gut microbiota were sourced from the Dutch Microbiome Project (DMP) (Lopera‐Maya et al. 2022). This study coordinated fecal metagenomic and genomic data from 7738 participants of European ancestry. Finally, we extracted GWAS data for 105 species‐level taxa. The GWAS summary statistics for the pan‐cancer phenotype was sourced from the UK Biobank Consortium. This study involved 422,659 individuals of European ancestry (ncase = 50,643 and ncontrol = 372,016). Considering the relatively low contribution of genetics to non‐melanoma skin cancer, we selected the GWAS data that exclude non‐melanoma skin cancer (accession numbers: ieu‐b‐4964, available from the IEU Open GWAS database [https://gwas.mrcieu.ac.uk/]). The GWAS summary statistics for dietary preferences can be publicly accessed from the GWAS Catalog (https://www.ebi.ac.uk/gwas/) (May‐Wilson et al. 2022). Dietary preferences involve the results of a food preference GWAS conducted on 161,625 European ancestry participants from the UK Biobank. The accession numbers for dietary preferences can be obtained through GCST90094687‐GCST90094873.

### 2.3 Selection of Genetic Instruments

For the genetic instruments of gut microbiota and dietary preferences, we set different significance thresholds (1 × 10^−5^ and 5 × 10^−8^, respectively). We performed a clumping procedure (*r* ^2^ < 0.001, window size = 10,000 kb) to exclude strong LD variants and ensure the independence of genetic instruments. We also excluded palindromic single nucleotide polymorphisms (SNPs). To ensure that the results were minimally affected by potential horizontal pleiotropy, we used LDlink (https://ldlink.nih.gov/?tab=home) to examine independent SNPs with an *r* ^2^ > 0.8 within a 50 kb range and excluded SNPs that affect smoking and type 2 diabetes. If present, we will exclude them in MR analysis. When matching outcome information, we also excluded SNPs with a minor allele frequency (MAF) < 0.01. To assess the strength of the genetic instruments, we calculated the *F*‐statistic using the formula: *F* = [(N−*k*−1)/*k*] × (*R* ^2^/1−*R* ^2^). Where N represents the sample size, *k* is the number of genetic instruments, and *R* ^2^ refers to the proportion of variance in the exposure explained by these genetic instruments. The formula for calculating *R* ^2^ is 2 × (1−MAF) × MAF × *β* ^2^. Genetic instruments with an *F*‐statistic < 10 were excluded. However, the GWAS data for dietary preferences lack MAF, so the *F*‐statistic is not calculated.

### 2.4 Statistical Analysis

#### 2.4.1 Genetic Correlation

Genetic correlation is commonly used to describe the shared genetic basis between complex traits and diseases (Bulik‐Sullivan, Finucane, et al. 2015; Bulik‐Sullivan, Loh, et al. 2015). The LMN genetic covariance matrix constructed from genome‐wide SNPs can be used to detect pathogenic variants of genotype SNPs LD. This enables the estimation of genetic correlation from prevalent SNPs representing all genetic variation and helps prioritize potential causal effects. *p* < 0.001 (0.05/50, after strict Bonferroni correction) is considered statistically significant. 0.001 < *p* < 0.05 is considered suggestive evidence.

#### 2.4.2 Causal Effect

Before causal inference, we estimated the *F*‐statistic of the genetic instruments to avoid biased estimates. Subsequently, we selected five methods (inverse variance weighted [IVW], MR Egger, Weighted median, Weighted mode, and Simple mode) to explore the complex causal network between the gut microbiota, dietary preferences, and pan‐cancer. When all genetic instruments are valid, IVW will provide decisive causal evidence. The results of IVW still require Bonferroni correction.

#### 2.4.3 Sensitivity Analysis

To examine the robustness and reliability of causal inference, sensitivity analysis is necessary. Cochran's Q test was conducted to identify the heterogeneity of genetic instruments. If *p* < 0.05, it indicates that causal inference is affected by heterogeneity, and random‐effects IVW is used to re‐estimate. The Egger intercept test was used to identify evidence of horizontal pleiotropy. If *p* < 0.05, it indicates that causal inference is affected by horizontal pleiotropy. We consider the result to be unrobust and exclude it. In addition to serving the same purpose as the Egger intercept test, MR‐pleiotropy residual sum and outlier (PRESSO) is also used to detect outliers. After removing outliers, we re‐estimated using IVW. Leave‐one‐out analysis will be used to further examine outliers. Scatter plots will help us visually detect outliers.

#### 2.4.4 Mediation Analysis

The effect size estimated by IVW will be used for the calculation of mediation effect and proportion. We used the causal steps approach to examine the diet preferences/gut microbiota/pan‐cancer mediation pathway. In addition, two‐step MR estimates the mediation effect and proportion. *β*_Mediation effect_ = *β*_Direct effect A_ × *β*_Direct effect B_. Mediation proportion (%) = *β*_Mediation effect_ ÷ *β*_Total effect_ × 100 (Figure 1).

### 2.5 R Software and Packages

All statistical analyses were performed using R (version 4.4.1). All analyses were performed using “ldscr” (version 0.1.0), “TwoSampleMR” (version 0.6.8) and “MRPRESSO” (version 1.0) packages.

## 3 Results

### 3.1 Genetic Correlations of Gut Microbiota on Pan‐Cancer

**FIGURE 2:**
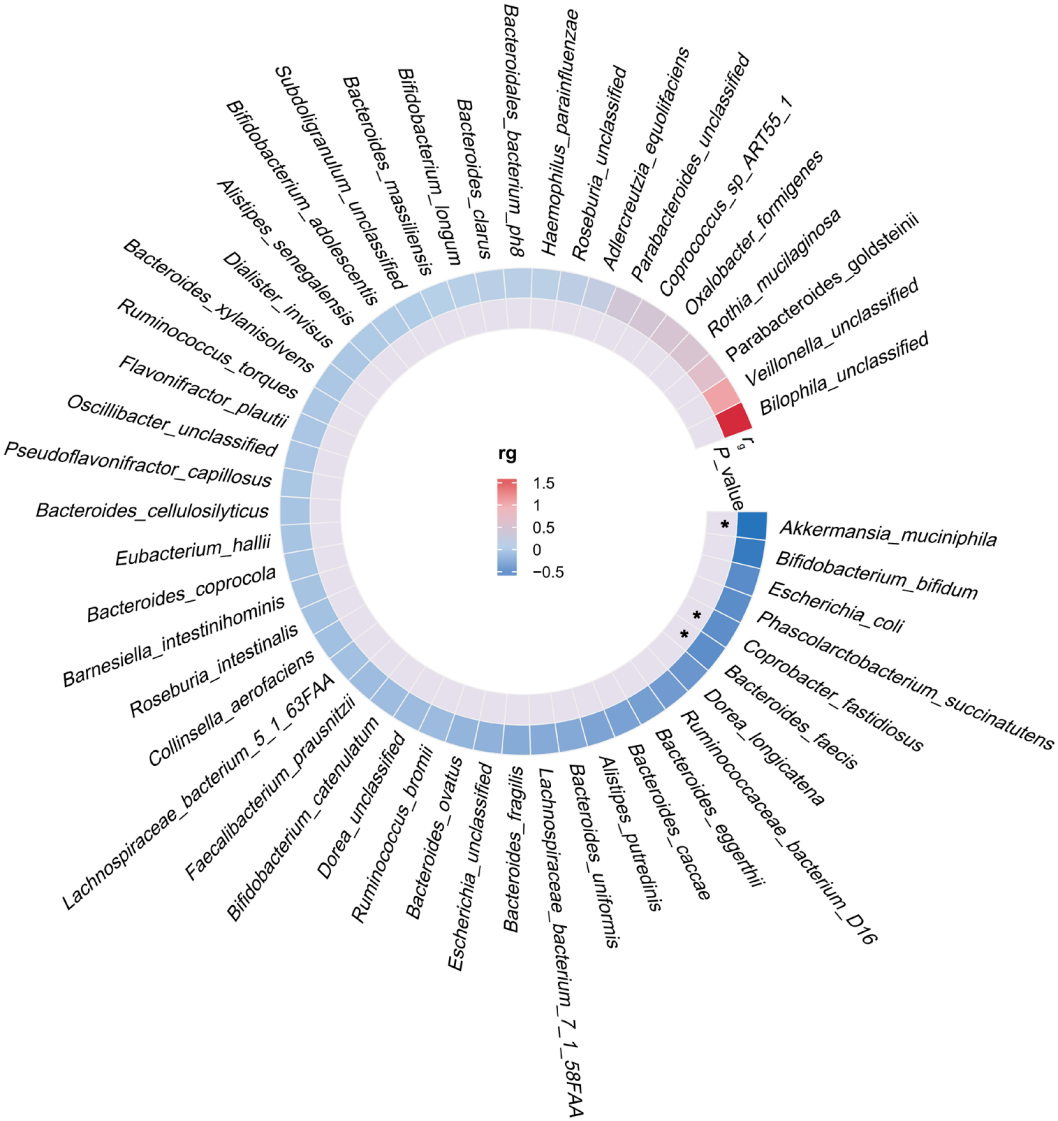
Circular heat map of the genetic correlations between gut microbiota and pan‐cancer. *p < 0.05.

The LDSC regression is used here to estimate the genetic correlation between the gut microbiota and pan‐cancer phenotype. Due to limitations such as low heritability, some taxa at the species level could not be used for the above analysis. Therefore, we conducted a genetic correlation assessment between 50 species and pan‐cancer phenotype (Figure 2). *Akkermansia muciniphila* (rg = −0.831, *p* = 0.032), *Coprobacter fastidiosus* (rg = −0.571, *p* = 0.014), and *Bacteroides faecis* (rg = −0.570, *p* = 0.039) exhibited suggestive genetic correlations with pan‐cancer phenotype (Table 1).

**TABLE 1:** The genetic correlations between gut microbiota and pan‐cancer.

Trait 1	Trait 2	*r*_g_	SE	*p*
*Akkermansia_muciniphila*	Pan‐cancer	−0.831	0.388	**0.032**
*Coprobacter_fastidiosus*	Pan‐cancer	−0.571	0.233	**0.014**
*Bacteroides_faecis*	Pan‐cancer	−0.570	0.277	**0.039**

*Note:* Bold: *p* < 0.05.

### 3.2 Causal Effects of Gut Microbiota on Pan‐Cancer

**FIGURE 3:**
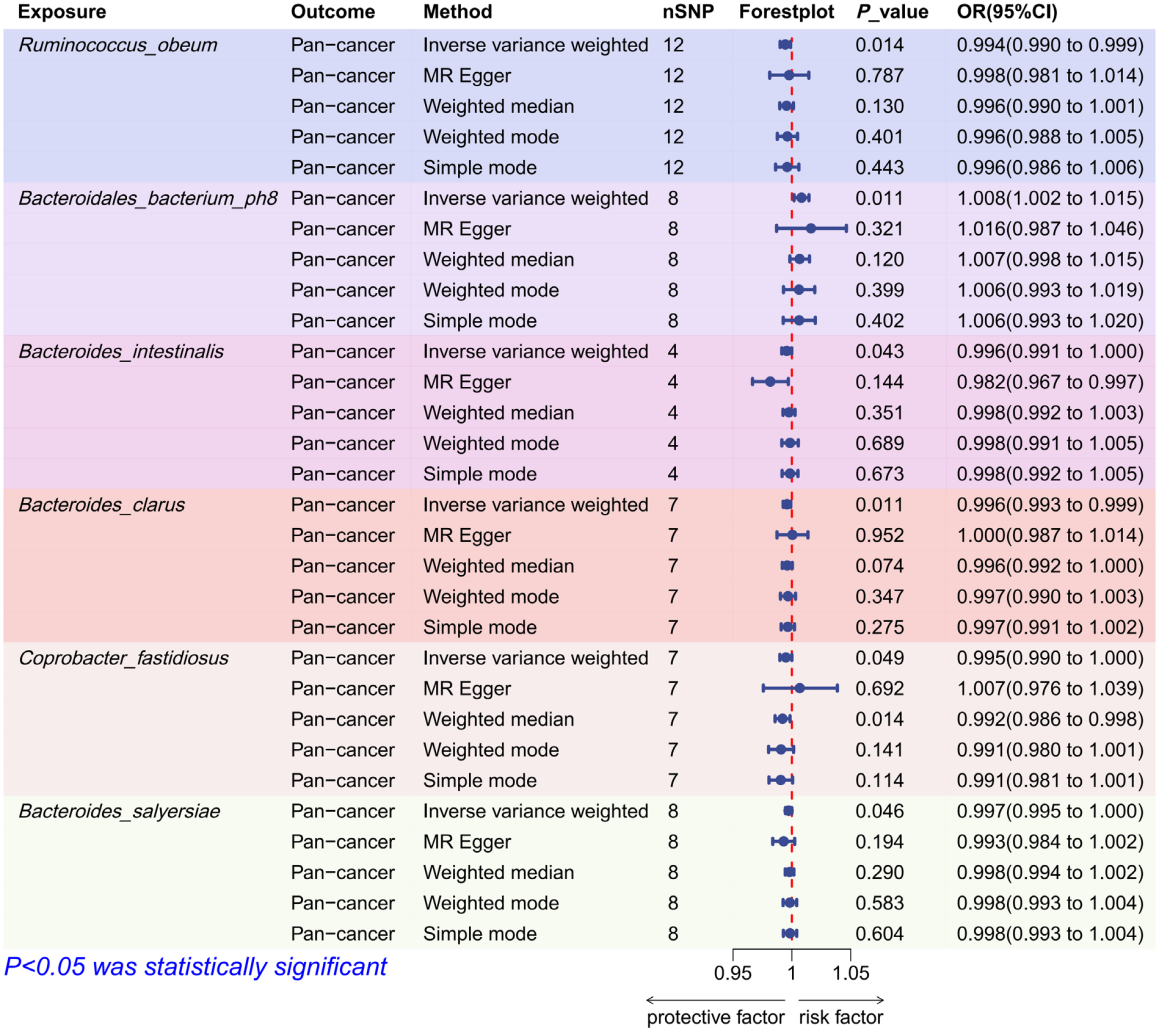
Forest plot of the causal effects of six gut microbiota on pan‐cancer. MR, Mendelian randomization; SNP, single nucleotide polymorphism.

A total of 976 genetic instruments meeting the criteria were included in the analysis (Table S2). The IVW was used to examine the potential causal effects of 101 species on pan‐cancer. *p* < 0.0005 (0.05/101, after strict Bonferroni correction) is considered a significant effect. 0.0005 < *p* < 0.05 is considered **a** suggestive effect. Finally, the IVW identified six suggestive causal effects (Figure 3). *Bacteroidales bacterium ph 8* is the only species genetically predicted to increase the risk of pan‐cancer (OR = 1.008, *p* = 0.011). In addition, *Ruminococcus obeum* (OR = 0.994, *p* = 0.014), *Bacteroides intestinalis* (OR = 0.996, *p* = 0.043), *Bacteroides clarus* (OR = 0.996, *p* = 0.011), *Coprobacter fastidiosus* (OR = 0.995, *p* = 0.049), and *Bacteroides salyersiae* (OR = 0.997, *p* = 0.046) were genetically predicted to reduce the risk of pan‐cancer.

**FIGURE 4:**
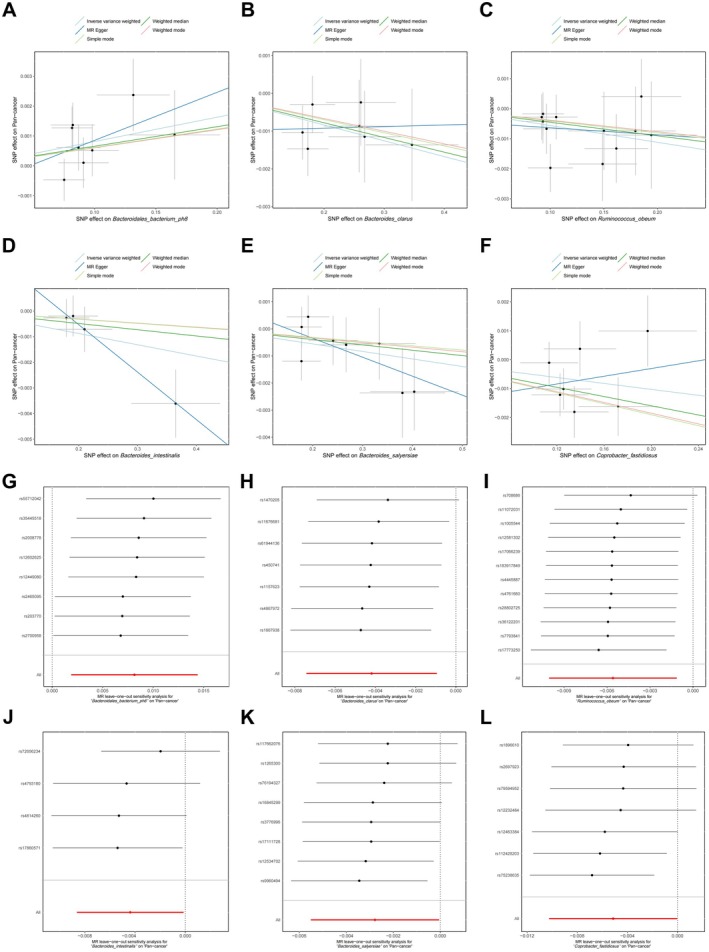
Scatter and leave‐one‐out plots of the causal effects of six gut microbiota on pan‐cancer. (A–F) Scatter plots of *Bacteroidales_bacterium_ph8*, *Bacteroides_clarus*, *Ruminococcus_obeum*, *Bacteroides_intestinalis*, *Bacteroides_salyersiae*, and *Coprobacter_fastidiosus*. (G–L) Leave‐one‐out plots of *Bacteroidales_bacterium_ph8*, *Bacteroides_clarus*, *Ruminococcus_obeum*, *Bacteroides_intestinalis*, *Bacteroides_salyersiae*, and *Coprobacter_fastidiosus*. MR, mendelian randomization; SNP, single nucleotide polymorphism.

Visual inspection of the scatter plot did not reveal any potential outliers (Figure 4A–F). The leave‐one‐out analysis further strengthened the above inspection (Figure 4G–L). Cochran's Q test did not provide evidence of heterogeneity (*p* > 0.05). The Egger intercept test and MR‐PRESSO did not find evidence of horizontal pleiotropy (*p* > 0.05). In addition, MR‐PRESSO did not detect the presence of outliers. The detailed results of the sensitivity analysis are shown in Table S3.

### 3.3 Causal Effects of Dietary Preferences on Pan‐Cancer

**FIGURE 5:**
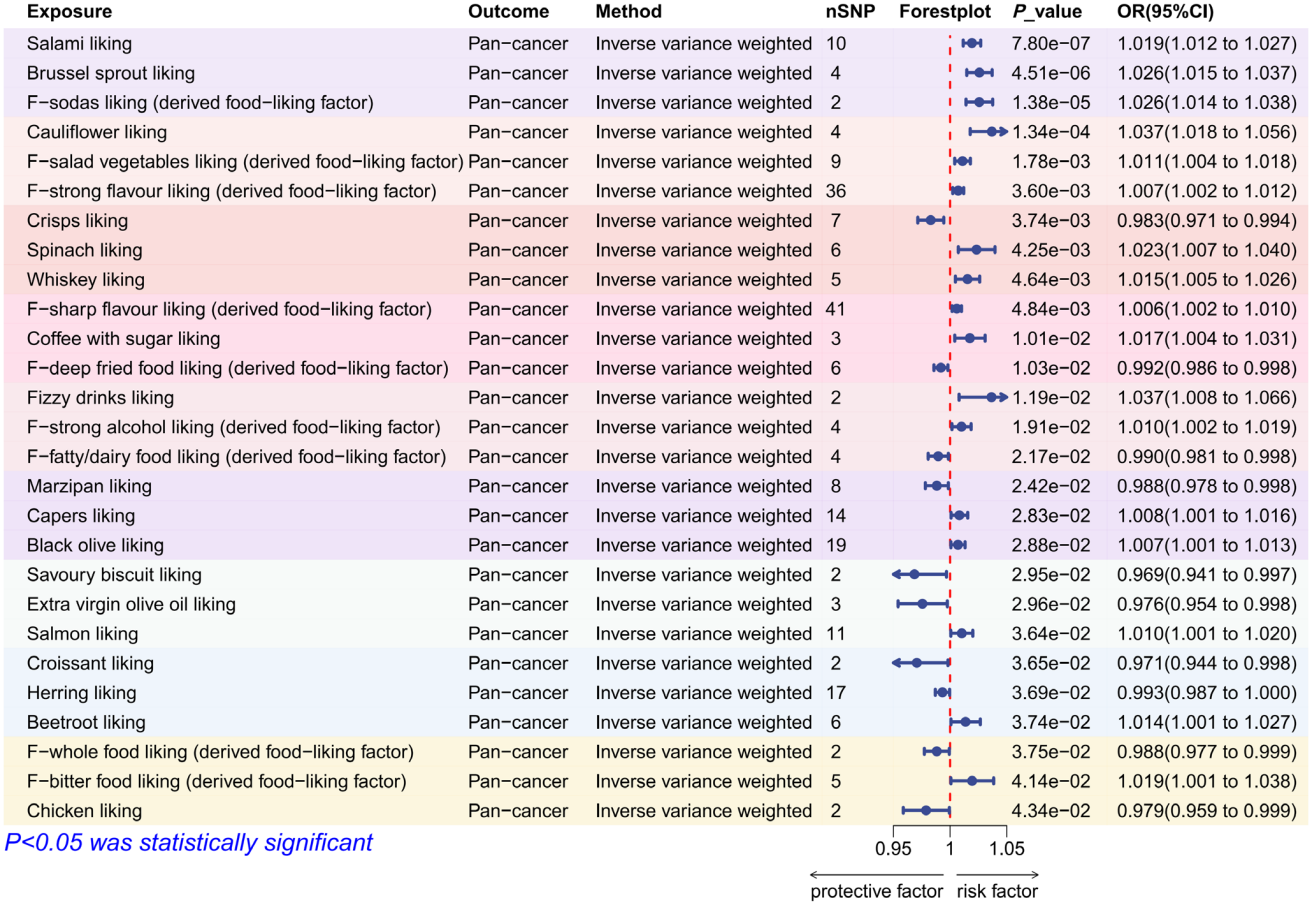
Forest plot of the causal effects of 27 dietary preferences on pan‐cancer. SNP, single nucleotide polymorphism.

A total of 1732 genetic instruments meeting the criteria were included in the analysis (Table S4). The IVW estimated the potential causal effects of 166 dietary preferences on pan‐cancer. *p* < 0.0003 (0.05/166, after strict Bonferroni correction) is considered a significant effect. 0.0003 < *p* < 0.05 is considered a suggestive effect. Finally, IVW identified four significant and 23 suggestive effects (Figure 5). Among them, the four significant dietary preferences are all associated with an increased risk of pan‐cancer. The detailed effect of different MR methods can be found in Table S5.

We applied the random‐effect IVW estimation to the effects showing heterogeneity as detected by Cochran's Q test. Some effects could not be estimated for horizontal pleiotropy due to an insufficient number of genetic instruments. Salmon liking was found to be affected by horizontal pleiotropy only by the Egger intercept test, while black olive liking was found to be affected by horizontal pleiotropy only by MR‐PRESSO. F‐strong flavor liking (derived food‐liking factor) was found to have horizontal pleiotropy by both methods. The effects of these three dietary preferences were not robust and were excluded from subsequent analyses. The detailed results of the sensitivity analysis are shown in Table S6.

### 3.4 Causal Effects of Dietary Preferences on Gut Microbiota

**FIGURE 6:**
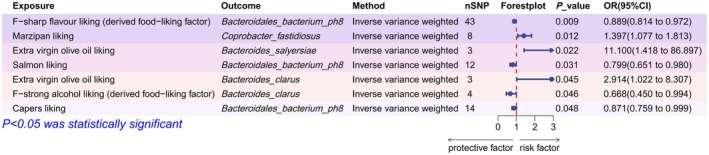
Forest plot of the causal effects of seven dietary preferences on four gut microbiota. SNP, single nucleotide polymorphism.

We further estimated the relationships between dietary preferences and gut microbiota. The IVW helped us identify seven dietary preference effects that regulate gut microbiota (Figure 6). The detailed effect of different MR methods can be found in Table S7.

We conducted extensive sensitivity analyses to highlight the robustness of the effects. The sensitivity analysis did not identify the presence of heterogeneity, horizontal pleiotropy, or outliers (Table S8). It is notable that the two effects of extra virgin olive oil liking could not be analyzed by MR‐PRESSO due to the insufficient number of genetic instruments.

### 3.5 Mediation Analysis

The causal steps approach was used to construct the network for dietary preferences/gut microbiota/pan‐cancer. Then, four pathways were identified (Table 2). The two‐step MR method was used to calculate the mediation effect and proportion. Extra virgin olive oil liking was found to reduce pan‐cancer risk by regulating *Bacteroides clarus* (Mediation effect *β* = 0.004, mediation proportion = 17.104%) and *Bacteroides salyersiae* (Mediation effect *β* = 0.007, mediation proportion = 28.884%). F‐strong alcohol liking (derived food‐liking factor) was found to drive an increase in pan‐cancer risk through *Bacteroides clarus* (Mediation effect *β* = 0.002, mediation proportion = 16.120%). Marzipan liking was found to reduce pan‐cancer risk through mediation by *Coprobacter fastidiosus* (Mediation effect *β* = 0.002, mediation proportion = 13.917%).

**TABLE 2:** The mediating effect of dietary preferences, gut microbiota, and pan‐cancer.

Mediator	Total effect *β* (95% CI)	Direct effect A *β* (95% CI)	Direct effect B *β* (95% CI)	Mediation effect *β*	Mediation proportion (%)
Extra virgin olive oil liking					
*Bacteroides_clarus*	−0.025 (−0.047, −0.002)	1.069 (0.022, 2.117)	−0.004 (−0.007, −0.001)	0.004	17.104
*Bacteroides_salyersiae*	−0.025 (−0.047, −0.002)	2.407 (0.349, 4.465)	−0.003 (−0.006, −0.000)	0.007	28.884
F‐strong alcohol liking (derived food‐liking factor)					
*Bacteroides_clarus*	0.010 (0.002, 0.018)	−0.403 (−0.800, −0.006)	−0.004 (−0.007, −0.001)	0.002	16.120
Marzipan liking					
*Coprobacter_fastidiosus*	−0.012 (−0.022, −0.002)	0.334 (0.074, 0.595)	−0.005 (−0.010, −0.000)	0.002	13.917

*Note:* “Total effect” indicates the effect of dietary preference on pan‐cancer, “Direct effect A” indicates the effect of dietary preference on gut microbiota, “Direct effect B” indicates the effect of gut microbiota on pan‐cancer. “Total effect”, “Direct effect A”, and “Direct effect B” were derived by inverse variance weighted.

Abbreviation: CI, confidence interval.

## 4 Discussion

To the best of our knowledge, this is the first study to systematically evaluate the genetic correlations and causal effects between the gut microbiota and pan‐cancer using GWAS summary statistics. Our study found suggestive genetic correlations between *Akkermansia muciniphila* , *Coprobacter fastidiosus*, and *Bacteroides faecis* with pan‐cancer. Moreover, the MR analysis identified six pairs of species‐level gut microbiota and pan‐cancer with suggestive causal effects. Dietary preferences also influence cancer risk. The MR analysis identified four dietary preferences with significant causal effects on pan‐cancer and suggestive causal effects for 23 dietary preferences. Meanwhile, the mediation analysis helped us find that the effects of certain gut microbiota on pan‐cancer are driven by dietary preferences. These findings will help us further understand the role of gut microbiota and dietary preferences in cancer and provide valuable insights into dietary patterns for populations at risk of cancer.

### 4.1 Gut Microbiota

In our findings, *R. obeum* showed a suggestive protective effect against pan‐cancer. *R. obeum* (It is now reclassified as *Blautia obeum* [*B.obeum*]) has been widely recognized as a probiotic. Metabolically associated fatty liver disease (MAFLD) and primary sclerosing cholangitis (PSC) are widely recognized as common causes of hepatocellular carcinoma (HCC) (Assis and Bowlus 2023; Powell et al. 2021). A metagenomic study found a significant reduction in the levels of *R. obeum* in the gut of MAFLD patients (C. Yang et al. 2023). The same changes have also been observed in patients with PSC (Kummen et al. 2021). In addition, colitis is considered a major risk factor for colorectal cancer (CRC) (Gros and Kaplan 2023; Rogler 2014). A study indicated that the absence of the *Ruminococcus* genus leads to increased susceptibility to dextran sodium sulfate (DSS)‐induced colitis (Zenewicz et al. 2013). *R. obeum* has also shown the ability to inhibit the proliferation of certain carcinogenic pathogens (such as *Clostridium perfringens* ) (Li et al. 2024; Hatziioanou et al. 2017). Our findings are consistent with earlier studies and suggest that *R. obeum* may reduce the risk of cancer and its associated pathogenic factors.

In our analysis, *B. bacterium ph 8* was the only gut microbiota species associated with risk in pan‐cancer. In a hypoxic and immunosuppressive tumor microenvironment, the *Bacteroidales* order was found to be enriched in pancreatic cancer tissues (Y. Chen et al. 2022). A meta‐analysis found that the *Bacteroidales* order was present at a higher proportion in prostate cancer patients (Huang et al. 2024). It is notable that there is still a lack of definitive studies showing the relationship between *B. bacterium ph 8* and cancer.

MR analysis found that three species under the *Bacteroides* genus indicated a protective effect against pan‐cancer. However, one study reported that a high abundance of *B. intestinalis* is associated with immunotherapy‐related colitis (Andrews et al. 2021). Immune‐related adverse events are often associated with excessive immune response. This suggests the pro‐inflammatory characteristics of *B. intestinalis*. In mouse experiments, *B. intestinalis* can degrade complex arabinoxylans in dietary fiber and release ferulic acid to enhance the Th1‐type immune response (Yasuma et al. 2021). Furthermore, obesity is a risk factor for the development of cancers (Avgerinos et al. 2019; Iyengar et al. 2016). Supplementation with *B. intestinalis* can significantly prevent diet‐induced obesity and hyperlipidemia (T. Wang et al. 2022). *Bacteroides* species, including *B. clarus* and *B. salyersiae*, are capable of degrading complex polysaccharides through polysaccharide utilization loci (PULs), providing energy and nutrients to the host (Zafar and Saier 2021). This metabolic capability helps maintain the stability and diversity of the gut microbiota. Gut microbiota dysbiosis has been found to be prevalent in cancer patients (Han et al. 2024; Y. Wang et al. 2024). In addition, a mouse study found that *B. salyersiae* can increase the fecal abundance of various anti‐inflammatory metabolites and probiotics (Dai et al. 2024). These studies highlight the beneficial interactions between the *Bacteroides* genus and a wide range of probiotics, as well as the reduction in the risk of pan‐cancers.

The current research on the relationship between *C. fastidiosus* and cancer is limited. The MR study has found that *C. fastidiosus* may reduce the risk of inflammatory bowel disease (IBD) (Zheng et al. 2024). Early studies have indicated that patients with IBD have a significantly increased risk of developing CRC (Rajamäki et al. 2021; Shah and Itzkowitz 2022). In our study, the similar results from LDSC and MR provide strong evidence for the negative correlation between *C. fastidiosus* and pan‐cancer. Future research should further explore the mechanisms by which *C. fastidiosus* and its metabolites regulate cancer risk.

### 4.2 Dietary Preferences

After rigorous correction, four significant dietary preferences were found to be associated with pan‐cancer. Meanwhile, these four dietary preferences all contribute to an increased cancer risk. Salami, as a processed meat, typically contains higher levels of fat, salt, and nitrates. Early studies have indicated that the high salt and nitrate levels in processed meats significantly increase the risk of gastric cancer, lung cancer, breast cancer, and other types of cancer (De Stefani et al. 2012; Wolk 2017). However, the preference for brussels sprouts, sodas, and cauliflower goes against their commonly known health benefits when it comes to cancer risk. This requires further research to clarify this interesting result.

Through mediation analysis, three dietary preferences were found to drive cancer risk by regulating the gut microbiota. A prospective study in Italian adults found that olive oil consumption is associated with a lower risk of cancer (Ruggiero et al. 2024). Chronic inflammation is an important driving factor in the development of cancer. However, extra virgin olive oil is rich in triterpenes, monounsaturated fatty acids, and various polyphenolic compounds (Moral and Escrich 2022). These substances are closely related to antioxidant properties. They often reduce the generation of reactive oxygen species and neutralize potential carcinogenic metabolites (Gorzynik‐Debicka et al. 2018). In addition, marzipan liking also produced similar results. The almonds in marzipan are also rich in monounsaturated fatty acids and vitamin E, which may help reduce cellular oxidative stress, thereby lowering the risk of DNA damage and mutations (Bojková et al. 2020; C. S. Yang et al. 2020). It is important to note that marzipan typically contains high levels of sugar, and excessive sugar intake may lead to obesity and metabolic disorders. Thus, consuming marzipan in moderation is key. About 4% of cancers worldwide are caused by alcohol consumption (Rumgay, Shield, et al. 2021). In 1988, the International Agency for Research on Cancer (IARC) classified alcoholic beverages as a Group 1 carcinogen. A recent review study indicated that alcohol and its metabolites can damage DNA and methylation repair from both genetic and epigenetic perspectives (Rumgay, Murphy, et al. 2021). Overall, the mechanisms of dietary preferences on cancer risk have been extensively studied. Future research should further explore the mediating role of the gut microbiota, an emerging concept.

The diversity and composition of the gut microbiota are regulated by various exogenous and endogenous factors. However, diet remains one of the key factors determining the status of the gut microbiota (Zmora et al. 2019). This also indicates that the interaction between diet and gut microbiota plays an important role in the outcome of health (Perler et al. 2023). The analysis of the gut microbiota regarding extra virgin olive oil liking found that genetic prediction significantly increases the relative abundance of two species under the *Bacteroides* genus. A previous study indicated that the consumption of extra virgin olive oil leads to enhanced growth of the *Bacteroides* genus (Barber et al. 2023). This is consistent with our results. Our study also found a significant negative correlation between strong alcohol liking and *B. clarus*. A study based on esophageal 16S rRNA data from alcoholic and non‐alcoholic esophageal cancer patients indicated that the abundance of the *Bacteroides* genus is significantly lower in alcohol drinkers (Rao et al. 2021). In addition, it has been reported that the proportion of the *Bacteroides* genus is particularly low in alcohol‐sensitive mice (Ferrere et al. 2017). The alcohol sensitivity significantly influences the regulation of alcohol's effects on health outcomes. This suggests that supplementation with the *Bacteroides* genus may help reverse the increased disease risk associated with alcohol consumption. There is a significant positive correlation between marzipan and *C. fastidiosus*. However, no current studies have shown a correlation between the two. We believe this may be related to the fiber content in marzipan. *C. fastidiosus*, as a species of the *Bacteroidetes* phylum, can participate in fiber fermentation to produce short‐chain fatty acids for energy. Further research is still needed to explore the interaction between marzipan liking and *C. fastidiosus* .

### 4.3 Advantages and Limitations

The gut microbiota and dietary preferences have currently attracted widespread interest and attention, especially in the field of oncology and dietetics. However, traditional research methods on the gut microbiota and dietary preferences are often influenced by various confounding factors. MR analysis using existing GWAS summary statistics can effectively eliminate the influence of confounding factors. Meanwhile, mediation analysis can further connect the gut microbiota and dietary preferences. This could help researchers clarify the dietary preference‐gut microbiota‐disease pathway.

However, we still acknowledge that there are some limitations in the study. Due to factors such as the small sample size, the study exhibits considerable heterogeneity, which was also observed in the Cochran's Q test. This also led to the LDSC analysis being affected by low heritability. Secondly, the GWAS data included in the study all originate from European ancestry, which limits the generalizability of our results to other populations. Finally, in order to obtain more comprehensive results, we chose to relax the significance threshold for the genetic instruments of the gut microbiota to 1e‐05. Although this is a commonly used threshold (Cui et al. 2023; Jiang et al. 2024), it may still increase the probability of false positives.

## 5 Conclusions

Overall, this study identified six species‐level gut microbiota and 24 dietary preferences with potential causal effects on pan‐cancer. Mediation analysis found that the effects of some gut microbiota are driven by dietary preferences. These findings will help us further understand the role of gut microbiota and dietary preferences in cancer and provide valuable insights into dietary patterns for populations at risk of cancer.

## Author Contributions

**Yuhang Zhou:** conceptualization (equal), data curation (equal), formal analysis (equal), resources (equal), software (equal), visualization (equal), writing – original draft (equal), writing – review and editing (equal). **Yun Feng:** conceptualization (equal), data curation (equal), formal analysis (equal), writing – original draft (equal). **Yue Wang:** conceptualization (equal), data curation (equal), formal analysis (equal), writing – original draft (equal). **Yiwen Wang:** conceptualization (equal), data curation (equal), formal analysis (equal), writing – original draft (equal). **Xiaolin Liu:** data curation (equal), formal analysis (equal). **Tao Sun:** funding acquisition (equal), supervision (equal), writing – original draft (equal), writing – review and editing (equal). **Junnan Xu:** funding acquisition (equal), supervision (equal), writing – original draft (equal), writing – review and editing (equal).

## Ethics Statement

The study used publicly available GWAS summary statistics and conducted secondary analyses of these data. Each GWAS was approved by its respective ethics committee.

## Conflicts of Interest

Author X.L. is employed by Liaoning Kanghui Biotechnology Co. Ltd. The remaining authors declare no conflicts of interest.

## Supporting information


**Data S1.** fsn370708‐sup‐0001‐DataS1

## Data Availability

Publicly available datasets were analyzed in this study, and all the details are provided in the manuscript. For additional information or queries, interested parties are encouraged to reach out to the corresponding author.
